# Evaluation of Physicochemical Properties of South African Cashew Apple Juice as a Biofuel Feedstock

**DOI:** 10.1155/2015/764196

**Published:** 2015-08-04

**Authors:** Evanie Devi Deenanath, Karl Rumbold, Michael Daramola, Rosemary Falcon, Sunny Iyuke

**Affiliations:** ^1^School of Chemical and Metallurgical Engineering, University of the Witwatersrand, 1 Jan Smuts Avenue, Braamfontein, Johannesburg 2000, South Africa; ^2^School of Molecular and Cell Biology, University of the Witwatersrand, 1 Jan Smuts Avenue, Braamfontein, Johannesburg 2000, South Africa

## Abstract

Cashew apple juice (CAJ) is one of the feedstocks used for biofuel production and ethanol yield depends on the physical and chemical properties of the extracted juice. As far as can be ascertained, information on physical and chemical properties of South African cashew apple juice is limited in open literature. Therefore, this study provides information on the physical and chemical properties of the South African cashew apple juice. Physicochemical characteristics of the juice, such as specific gravity, pH, sugars, condensed tannins, Vitamin C, minerals, and total protein, were measured from a mixed variety of cashew apples. Analytical results showed the CAJ possesses specific gravity and pH of 1.050 and 4.52, respectively. The highest sugars were glucose (40.56 gL^−1^) and fructose (57.06 gL^−1^). Other chemical compositions of the juice were condensed tannin (55.34 mgL^−1^), Vitamin C (112 mg/100 mL), and total protein (1.78 gL^−1^). The minerals content was as follows: zinc (1.39 ppm), copper (2.18 ppm), magnesium (4.32 ppm), iron (1.32 ppm), sodium (5.44 ppm), and manganese (1.24 ppm). With these findings, South African CAJ is a suitable biomass feedstock for ethanol production.

## 1. Introduction

Cashew tree is a tropical and subtropical tree belonging to the family Anacardiaceae, the genus* Anacardium* Linn, and the species* Anacardium occidentale* Linn var.* nanum* [1–3]. Cashew tree is a branched, evergreen tree with a height between six meters (6 m) and twelve metres (12 m) and diameter between four meters (4 m) and twelve metres (12 m). Cashew trees grow in regions characterized by altitude of 0–1000 m, average annual temperature from 17°C to 38°C and average annual rainfall from 500 mm to 3,500 mm [4]. Furthermore, cashew tree is a native tree of north and south America and is believed to have originated from the* cerrados* of central Brazil and the* restinga* of northeastern Brazil and then spread to a number of other regions such as Cambodia, Gambia, India, Indonesia, Kenya, Malaysia, Mozambique, Myanmar, Philippines, Sri Lanka, Sudan, Tanzania, Thailand, Uganda, and Vietnam during the fifteenth and sixteenth century by Portuguese and Spanish explorers [3–6]. The cashew nut and cashew apple (CA) are the two morphological parts of interest from the cashew tree. The cashew nut and CA are the fruits of the cashew tree, with the nut referred to as the true fruit and the apple referred to as the false fruit. The cashew nut is kidney-shaped with an exterior, hard shell and interior white kernel. The CA is a hard, pear-shaped, and green fruit that turns red, yellow, or orange during maturation [7–10].

The CA is rich in sugar, riboflavin, Vitamin C, iron, tannins, minerals, and organic acids [1, 2, 11]. As the cashew nut grows the CA grows, with the average weight of the apple about 5 to 10 times larger than the nut [12–14]. During initial growth stages, the CA growth is slow but eventually swells and develops into a fleshlike fruit at the base of the nut during the final growth stages [4, 15]. Economically, the cashew nut is the main commercial product and is the worlds' second largest trade nut after almonds, whilst a small amount of products are derived from the CA [2, 16, 17]. The CA is usually a neglected product and, if utilized, the CA derived products are fruit juices, vinegar, jam, syrups, and cashew wine [1, 2, 7, 8, 11, 18]. In 2006, the estimated worldwide CA production was 30 million metric tons with the leading CA producing regions being Vietnam (8.4 million tons), Nigeria (5 million tons), India (4 million tons), Brazil (1.6 million tons), and Indonesia (1 million tons) [2, 11, 17]. Other than food and industrial products, cashew tree contributes to fodder, fibre, timber, inks, insect repellent, and medicine [4, 15].

The CA and cashew apple juice (CAJ) have numerous industrial applications. Examples of these applications are (a) sugar separation from CAJ, (b) beverage production, (c) evaluation of yeast strains for ethanol and sugar tolerance, (d) detection of antioxidant properties, (e) improving nutritional quality of other fruit juices, and (f) production of vinegar, lactic acid, biosurfactants, dextransucrase, oligosaccharides, and fuel ethanol [1, 9, 10, 12, 13, 18, 20, 23–34]. For sugar separation, pure glucose and fructose can be extracted from CAJ by adsorptive chromatography and used for the production of syrups. This type of syrup is an alternative to the present syrup supply from corn hydrolysis [25]. Cashew wine, an alcoholic beverage, is produced from the fermentation of CAJ and in Goa, India, it is referred to as* fenni* [20]. For cashew wine production, the CAJ is fermented using* S. cerevisiae* yeasts at temperatures between 28°C and 30°C and pH of 4.0. Alcohol content between 6% (v/v) and 10.6% (v/v) is possible [12, 33]. Yeast tolerance of yeast species isolated from fermenting CAJ was determined in a study by Osho [28]. Seventeen* Saccharomyces* strains were isolated and four of these strains, namely,* S. cerevisiae* strains 0271, 0269, and 0260 and* S. uvarum* 0275 show alcohol tolerance ranging from 9% (v/v) to 12% (v/v). These yeasts are also able to grow in various glucose concentrations from 10% (w/v) to 25% (w/v) and respond well to osmotic stress [28]. Anacardic acids, which are an alkyl phenol of the CA, possess antioxidant activity by inhibiting superoxide anion and xanthine oxidase [1]. This acid has been shown to have a greater antioxidant capacity than trolox, salicyclic acid, and hydroxytyrosol [1]. Additionally, the phytyl side chain of anacardic acid can be used as a Vitamin E substitute in body products [1]. The main nutritional component of CA is Vitamin C. It was found that CA has a greater concentration of Vitamin C than other fruits such as oranges, grapes, mangoes, lemons, and pineapples [13]. The CAJ was blended with orange, pineapple, grape, and mango fruit juices to increase the Vitamin C concentration of these commercial fruit juices [13]. The flavour properties of the alternate juices were accepted by consumers and thus CAJ can be mixed with other fruit juices to improve the Vitamin C quality [13]. Vinegar, as a fermentation product from CAJ, is useful in the food industry. The vinegar product as a result of fermentation is a natural product rather than a synthetic product. This natural vinegar is a rich source of amino acids acquired from the fruit juice [18]. The process of vinegar production from CAJ involves the action of* S. cerevisiae* yeasts on the sugary juice substrate to ethanol, followed by the conversion of the ethanol by acetic acid bacteria to produce acetic acid or vinegar [18]. The production of lactic acid from fermentation of CAJ is of interest, as lactic acid can be used in the preservation of food products, especially dairy products and in the manufacture of polylactic acid biopolymers for food packaging [31]. For lactic acid fermentation, the CAJ is fermented by the action of lactic acid bacteria (LAB) such as* Lactobacillus bulgaricus, Lactobacillus leichmannii, Lactobacillus delbrueckii, Lactobacillus amylophilus*, and* Lactobacillus plantarum*. A lactic acid yield of 95% (2.3 g/L/h) is possible under fermentation conditions of temperature = 37°C, pH = 6.5, and sugar concentration = 50 g/L [31]. Another application of CAJ fermentation is for the production of biosurfactants [9, 34]. Surfactants are used in household detergents and are derived from petrol. Biosurfactants, on the other hand, are alternative products that are derived from plant biomass or vegetable oils by the use of MOs [9, 34]. Structurally, biosurfactants are made up of hydrophilic and hydrophobic components. The hydrophilic component consists of amino acids and polysaccharides and the hydrophobic component consists of lipids [34]. The structure develops from the type of material and processing conditions. This structure enables the biosurfactant molecule to function as emulsifying, foaming, and detergent agents [9, 34]. There is a market for biosurfactants as they are less toxic products and are environmentally safe and degradable; however due to high costs there is minimal production and usage of biosurfactants [9, 34]. For this reason, Rocha et al. [9] demonstrated the use of CAJ as a less expensive substrate to provide a biosurfactant. CAJ was fermented at a temperature of 30°C and pH of 7.0, with the action of* Acinetobacter calcoaceticus* to produce an emulsion biosurfactant. The study revealed the microorganism (MO) produced the biosurfactant as a by-product during the stationary growth phase, when the sugar concentration decreased. The emulsifying activity was found to be 58.8% [9]. Other than biosurfactants, CAJ as a fermentation medium can be used to produce dextransucrase and oligosaccharides dextransucrase can be used as a food preservative and oligosaccharides can be used as a prebiotic [23, 24, 26, 27, 32]. Dextransucrase is an enzyme produced by the LAB,* Leuconostoc mesenteroides*. This enzyme is a glycosyltransferase that catalyses reactions of glycosyl residues between donor and acceptor molecules to produce fructose, mannitol, dextran, oligosaccharides, and polymers [23, 24, 26, 27, 32]. For dextransucrase production, the CAJ medium was supplemented with sucrose, yeast extract, and dipotassium hydrogen phosphate followed by fermentation at a temperature of 30°C, pH of 6.5, and LAB strain* L. mesenteroides* B-512F [23]. The study showed that high dextransucrase activity and microbial growth were possible and hence CAJ is a useful substrate for the production of dextransucrase and the supplementation of the CAJ with other nutrients initiates the enzyme production [23]. Additionally, LAB such as* L. citreum* B-742 and* L. mesenteroides* B-742 can be used for dextransucrase production and fructose which is released can be further reduced to mannitol. Mannitol can be used as a sweetener or “texting agent” [24] in food products [24, 26, 32]. For the production of oligosaccharides, sucrose is also added to the CAJ and the native sugars of CAJ, which are glucose and fructose, serve as acceptor molecules during enzyme reactions to produce oligosaccharides [24, 26, 27]. CAJ fermentation conditions to produce oligosaccharides were temperature = 32°C and pH = 6.7. The concentration range of oligosaccharides was 1.80 g/L to 9.30 g/L and additionally mannitol was produced with a concentration range of 5.60 g/L to 17.44 g/L [24]. The use of CAJ for in biofuels is relatively new and simple procedures are employed to obtain ethanol. In general, CAJ is extracted from CAs and the monosaccharide sugars, namely, glucose and fructose are fermented by yeasts. First, CAJ is pretreated by adding gelatine powder for the precipitation of tannins. The CAJ is then filtered or centrifuged and sterilized for fermentation [29, 30]. Pinheiro et al. [29] showed that 44.40 g/L of ethanol can be produced and Neelakandan and Usharani [30] showed that 9.35% (v/v) of ethanol was possible using immobilized yeast cells.

The aforementioned information lucidly indicates the potential applications of CAJ for industrial applications. One of the potential applications, not fully exploited in South Africa, is the use of the CAJ as feedstock for ethanol production as a type of biofuel. Obtaining high yield of bioethanol with CAJ as the feedstock depends on many factors, amongst which is the physicochemical characteristics/nature of the CAJ. For instance, a recent study showed this CAJ yields a high concentration of ethanol [35]. Against this background, this study focused on physicochemical characterization of South African CAJ with an aim of documenting it as a biomass feedstock, particularly in South Africa. The CAJ used in the study was an extract of cashew apples grown and harvested in the KwaNgwanase region located in the KwaZulu-Natal (KZN) province of South Africa.

## 2. Materials and Methods

### 2.1. Raw Materials

Cashew apples were collected from a Coastal Cashews plantation site located in KZN (legal name: Dotcom Trading 25; P.O. Box 330, KwaNgwanase, 3973 KwaZulu-Natal, South Africa). The area is also known as Maputaland, Kosi Bay, or rural Manguzi. The Coastal Forest Reserve is the main region on the east coast of sub-Saharan Africa where cashew trees grow and the reserve stretches for approximately 18 km along the northeast KwaZulu-Natal coastline in South Africa to Mozambique. The Coastal Cashews plantation is 1000 hectares in size and is the only cashew tree plantation in South Africa. CAs of a mixed variety were collected in February from the plantation in 2011 for this study.

### 2.2. Methods

#### 2.2.1. Extraction of Cashew Apple Juice

**Figure 1:**
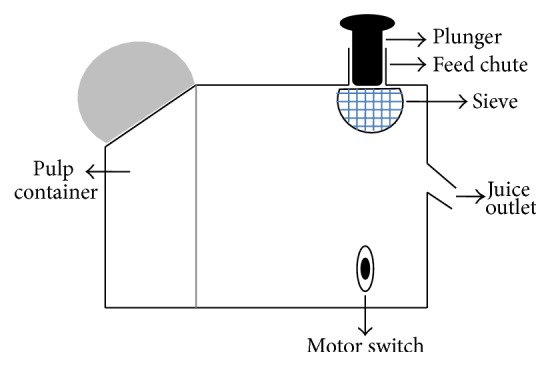
A schematic diagram representing the KENWOOD fruit processor used for CAJ extraction.

CAJ was extracted from CAs using a food processor (KENWOOD-JE720). The extraction was done at the Cashew Nut factory on the Coastal Cashew plantation. The schematic diagram shown in Figure 1 represents the fruit processor used for the juice extraction. The juice extraction procedure was as follows: (1) the motor was switched ON and a collection jug was placed at the juice outlet, (2) sliced CAs were added to the feed chute, until full, (3) the plunger was used to push the CAs down the feed chute, (4) the extracted juice passed through the stainless steel sieve and flowed through the juice outlet into the collection jug (kept on ice), whilst the pulp accumulated in the pulp container, (5) as the extraction continued the pulp container was emptied when necessary, and the pulp was discarded, and (6) when the collection jug was full the CAJ was transferred into recapable polyethylene bottles. The bottles with the CAJ were frozen to preserve the quality of the juice prior to analysis.

#### 2.2.2. Physicochemical Characterisation of the CAJ

Physicochemical characteristics investigated for the CAJ were specific gravity, pH, sugar content, condensed tannins content, Vitamin C content, minerals content, and total protein content. Prior to the characterization, the frozen CAJ was thawed at 4°C for approximately 18 hrs, followed by centrifugation (BECKMAN model TJ-6) at 3,500 rpm for 10 minutes to remove residual pulp that may have passed through the sieve during extraction. The pulp was discarded. A clarified, free-flowing CAJ was used for the characterization and each analysis/measurement was repeated to ensure accuracy and reproducibility of results.

Specific gravity of the juice was measured directly using a hydrometer (model: BREWMAKER Limited, National Food Products, Johannesburg, South Africa) and the pH was determined using the Hamilton pH sensor (model: EF-set 12/200/2K8, Labotec (Pty) Ltd., Johannesburg, South Africa).

Total sugar content of the CAJ was analysed with a High Performance Liquid Chromatography (HPLC model: LC 1100 series, Agilent Technologies Inc., Johannesburg, South Africa) equipped with a solvent delivery system (quaternary pumps), autosampler, refractive index, wavelength detectors, thermostated column compartment, and ChemStation software programme. For the sugar analysis, the refractive index detector (RID) and the Aminex Fermentation Monitor (AFM) column (BIORAD) were used. Conditioning of the HPLC during the analysis was according to Table 1. Prior to the analysis, the HPLC was calibrated using external standards of glucose, fructose, and maltose sugar at a concentration of 2% (w/v).

Content of the condensed tannins of the CAJ was measured using the Vanillin-HCl method as discussed elsewhere [19, 36, 37]. Determination of the content of Vitamin C of the CAJ was by iodine titration described by Lowor and Agyente-Badu [19], after a few modifications to the procedure. To determine the unknown Vitamin C concentration of the CAJ sample, standard ascorbic acid solutions of known concentrations were prepared and a standard curve was constructed. The concentrations of the standard solutions were 0.5, 1, 1.5, and 2 gL^−1^ of ascorbic acid. The titration assay was carried out for each of the standards and CAJ sample. The volumes recorded for the standards were used to construct a standard curve of volume of titrant (mL) versus ascorbic acid concentration (gL^−1^). The volume recorded for the CAJ sample was used to determine the amount of ascorbic acid from the standard curve and the concentration was expressed as mg/100 mL.

For the analysis of minerals, CAJ was digested according to the method described by Lowor and Agyente-Badu [19]. Following digestion, the minerals present in the predigested CAJ sample were quantified by Atomic Absorption Spectroscopy (AAS) (model: Spectra AA 55B, Varian Australia (Pty) Ltd., 1996–2000) equipped with a flame ionization detector (red cone: 1 mm), mineral specific lamps, and air/acetylene gases. The analysis considered zinc, copper, manganese, magnesium, iron, and sodium as minerals of interest. Standards of each of the above mentioned minerals were prepared using 1000 ppm ICP and Atomic Absorption Spectroscopy Standards (SMM Instruments). The protein composition of the juice was determined by the Bradford Method, also known as the Coomassie Blue assay [38]. Experiments were carried out in triplicate and the protein composition expressed in gL^−1^.

Statistical analysis of the measurements was carried out as well using descriptive statistics such as the mean and standard deviations to ensure the validity of the results.

## 3. Results and Discussion

### 3.1. Collection of CAs

**Figure 2:**
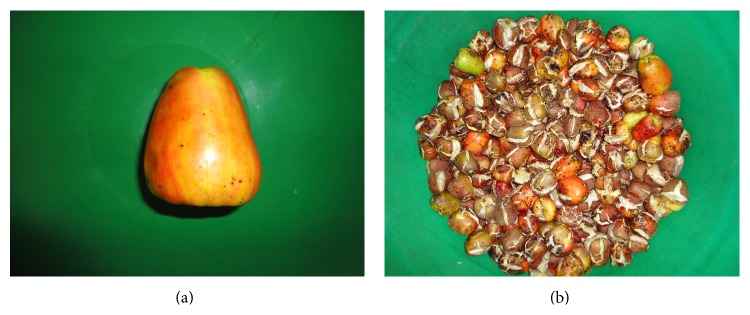
((a)-(b)) Pictures showing a single cashew apple and a mixed variety of cashew apples from the Coastal Cashew plantation in KwaNgwanase, South Africa.

The main cashew apple harvest season in South Africa is between January and April, when the average rainfall increases to 96% and approximately 380 tons of cashew apples is produced. During the February 2011 harvest season, CAs were collected from the cashew trees at the Coastal Cashew plantation. Pictures of samples of the CA are depicted in Figures 2(a) and 2(b).  Figure 2(a) shows a single CA that is yellow to orange in colour and Figure 2(b) shows a mixed variety of CAs such as red apples, green apples, partial red/yellow to orange apples, and reddish-brown apples. The cashew apples found at the base of the trees and around the plantation were collected. Additionally, apples that did not show cashew nut attachment or were of lesser quality were selected and collected. This method was used to prevent overlapping of market-value products such as harvesting and selling of cashew nuts and supplying of the apples for food consumption and/or manufacturing of CA-related products.

### 3.2. Physicochemical Characterisation

Tables 2 and 3 show the physical and chemical characteristics of the cashew apple juice compared with available results from literature. The specific gravity value shown in Table 2 was recorded at the point where the liquid intersected the hydrometer and the value is in agreement with the value reported by Akinwale [13], for CAJ extracted from Nigerian CAs, with a percentage difference of 0.38%. However, the value of specific gravity in this study is lower by 1.79% compared to the specific gravity reported by Attri [20], for CAJ obtained from CAs grown in Bengal (India). In addition, the pH obtained in this study corresponds to the pH range of 3 to 4, for normal fruit juices [38], and in agreement with the pH value reported by Akinwale [13] for Nigerian CAJ and Lowor and Agyente-Badu [19], for Ghanaian CAJ (Table 2). The pH values of CAJ from CAs grown in Benin are lower than the pH of CAJ in this study [17].

In Brazil, substantial amount of research was conducted on CAJ. Previous studies from this country revealed that the pH values of Brazilian CAJ ranged from 3.80 to 6.00 [8, 17, 22]. Comparison of the pH of Brazillian CAJ (from Rio de Janeiro) with South African CAJ (from KwaNgwanase) shows that pH of South African CAJ is higher than the pH of Brazilian CAJ (Table 2). In another study conducted in Brazil on the CAJ obtained from another region (Sao Paulo), the pH of the CAJ agrees with the pH value obtained for South African CAJ [8]. Physicochemical characterization of Indian CAJ has also been reported [20, 21]. In studies carried out on Indian CAJ from Bengal and Ariyalur District, it was reported that the pH values of the Indian CAJ were in agreement with the pH values reported for Brazilian CAJ but differ slightly from the pH values obtained in this study (Table 2). The specific gravity and the pH of the CAJ analysed in this study compare favourably with the specific gravity and the pH reported for CAJ from West Coast of Africa such as Nigeria and Ghana.

Furthermore, the inconsistency in the values of pH and specific gravity reported for CAJ from different countries could be attributed to difference in climatic conditions such as rainfall, wind, or sunlight prevailing in these countries. At the same time, effect of soil conditions such as soil composition and soil nutrients on the quality of CAs cannot be overlooked as well. Therefore, it could be assumed the climatic conditions in the KwaNgwanase area of KwaZulu-Natal, South Africa, are similar to the climatic conditions in West Coast of Africa.

The sugar concentrations quantitatively determined by HPLC showed glucose and fructose as the predominant sugars of CAJ (Table 3). The abundance of glucose and fructose is in agreement with previous studies based on studies from Brazilian cashew apples [23, 25–27]. Results from previous research studies on the characterization of CAJ indicate glucose concentrations range from 43.28 gL^−1^ to 46.34 gL^−1^ and fructose concentrations from 37.46 gL^−1^ to 56.00 gL^−1^ [23, 25–27]. The identification of glucose and fructose is consistent with previous studies; hence these are native sugars of CAJ and the difference in concentration is probably dependent on the CA variety. Additionally, maltose content was determined and the concentration was 2.18 gL^−1^. The detection of maltose for CAJ has not been previously reported but the presence of maltose is not unusual, as maltose is a sugar component of fruit juices [39].

The concentration of the condensed tannin in the South African CAJ was 55.34 mgL^−1^ (Table 3). This value is lower than the value reported by Lowor and Agyente-Badu [19]. Though pH and specific gravity of the South African CAJ are similar to those of Ghana CAJ, the content of tannin differs. Condensed tannins, as suggested by Lowor and Agyente-Badu [19], could be influenced by climate conditions, and drier climates contribute to a higher tannin content. From this information it can be assumed that the KwaNgwanase area is less dry than other CA growth regions. These condensed tannins, also known as proanthocyanidins, are phenolic compounds made up of oligomers and polymers of flavans which are mainly found in plant materials [37, 40, 41].

The content of Vitamin C obtained in this study is lower when compared to the Vitamin C content of CAJ from Ghana, Nigeria, and India (Table 3). The value is also similar to those of CAJ from Sao Paulo, Brazil (Table 3) [8, 13, 19, 20, 42]. Consequently, it could be deduced that CAJ characteristics are regional dependent. Vitamin C content is an important requirement if the CAs or the juice is consumed as food or used in the manufacture of food or medicinal products because Vitamin C provides nutritional value as well as medicine for treating diseases such as scurvy, gastritis, and rheumatism [8, 13, 19–22, 43].

The highest mineral contents for the CAJ were recorded for sodium and magnesium and the lowest for iron and manganese. These mineral compositions of the KwaNgwanase CAJ are similar to those of Ghana CAJ [19]. In the study documented by Lowor and Agyente-Badu [19], the characterisation of mineral results showed that sodium and magnesium possess the highest composition and iron the lowest. The results in this study are in accordance with the exception of the lowest mineral composition. In the present study, manganese showed lower composition than iron and this mineral was not previously investigated for CAJ. Other than iron and manganese, the zinc composition in the present study and previous study [19] was low which is common for fruit juices [43]. Similarities in mineral compositions are probably due to similar mineral deposits in the cashew tree cultivation areas in KwaNgwanase, South Africa, and Ghana.

The total protein value reported in this study is lower compared to the previous studies which reported a total protein content of 2.58 g/L (Table 3). These studies were based on CAJ characterization from CAs growing in Brazil [23, 24, 27].

**Figure 3:**
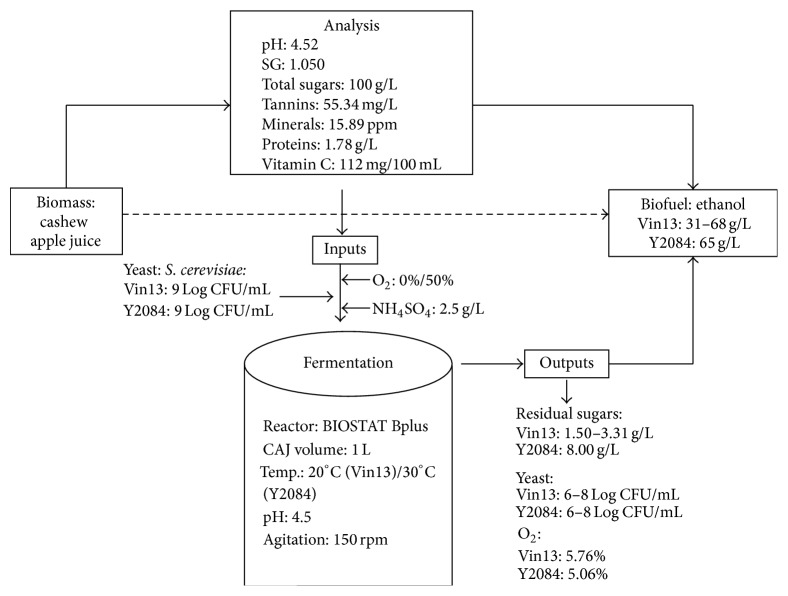
Cashew apple juice-ethanol production process.

Based on the characteristics, the CAJ to ethanol conversion is shown in Figure 3. This represents data from this study and from the findings of Deenanath et al. [35].

Overall, the specific gravity and pH reflect a prediction of the ethanol concentration and the acidity of the juice, respectively. These physical characteristics proved useful for ethanol production as investigated by Deenanath et al. [35]. From a specific gravity value of 1.050, 6% to 7% ethanol production is a predicted value. This coincides with the concentration obtained by fermenting this CAJ [35]. Additionally, the acidic nature of the juice (pH 4.52) is an ideal environment for yeast growth [35]. The presence of monosaccharide sugars in this particular feedstock is advantageous as it enables easy and fast assimilation by yeast cells, thus enhancing the yield of ethanol [35]. Tannins are known to reduce microbial contamination [40], and, for the purpose of ethanol production, the presence of these tannins can possibly prevent external microbial contamination which can compete for the sugars and nutrients of the CAJ and essentially lead to a decrease in the ethanol concentration. Vitamin C probably contributes to the acidic pH of the juice and can serve as a cofactor for microbial enzymatic reactions during ethanol fermentation [44–46]. The minerals are a source for yeast growth and proteins serve as a nitrogenous source for the yeasts. Based on the findings by Deenanath et al. [35], the concentrations of Vitamin C, minerals, and total protein are useful as high viability counts were reported during fermentation and thus yeast metabolism was optimal. CAJ as a type of biofuel feedstock consists of sugars in its simplest form and is thus readily available for fermentation by yeasts microbes, without pretreatment which is a necessity for lignocellulose feedstocks such as bagasse and wood chips. Other types of feedstocks comparable to CAJ are molasses from sugar beet and sugar cane as they are an abundance of sucrose [47, 48]. Recent studies on biofuel technology focused on fermenting sugar beet and cane molasses with immobilized yeasts with the intention to produce ethanol [47, 48]. Batch fermentation of sugar beet molasses (total sugars: 130 g/L) resulted in 60.36 g/L of ethanol with immobilized yeasts and 50.34 g/L with free yeast cells [47]. Sugar cane molasses (total sugars: 100 g/L) fermented in continuous reactor resulted in 44.06 g/L with immobilized yeasts and 27.90 g/L with free yeast cells [48]. Compared to ethanol concentration of CAJ (Figure 3), beet molasses-ethanol and cane molasses-ethanol are lower, with beet molasses being closer in ethanol concentration to CAJ (Figure 3) than cane molasses. It is important to note, in these studies [47, 48], immobilized yeasts were used to enhance the cell growth for higher ethanol concentrations, whereas in the study by Deenanath et al. [35] yeasts in their free form were able to produce ethanol efficiently; hence properties of South Africa CAJ feedstock stimulate and support yeast growth for ethanol fermentation.

## 4. Conclusion

Physicochemical characteristics of the South African cashew apple juice (CAJ) have not been previously documented and the aim of this study was to characterise CAJ growing in South Africa, with a view to encouraging the use of CAJ as a biofuel feedstock. Comparison of these results with literature suggested that the results are in agreement with previous studies, with some variations. In addition, the results of the study confirm that difference in climatic conditions causes variation of the physicochemical characteristics of the CAJ. The South African CAJ possesses high sugar content, making it ideal as a feedstock for biofuels to produce ethanol by fermentation. Furthermore, the condensed tannins present in the CAJ will prevent contamination of the sugary content of the feedstock to achieve maximum ethanol yield. Abundant concentration of Vitamin C, a variety of minerals, and the presence of proteins will serve as nutrients for the growth of the microorganism growth applied for ethanol production. This study showed that South African CAJ encompasses suitable feedstock properties for biofuels. Furthermore, for this research, cashew apples that did not show cashew nut attachment and the apples that naturally fell from the trees were collected for usage; hence this will not affect the possible production of other products. The presence of a suitable and relatively cheap (ZAR30.00/kg) feedstock, such as CAJ, could promote sporadic growth of the biofuel industry in South Africa and usage will prevent discarding of the product.

## Figures and Tables

**Table 1:** Conditioning of HPLC for sugar analysis.

Parameter	Values
Mobile phase (sulphuric acid)	0.001 M
Flow rate	0.8 mLmin^−1^
Column temperature	60°C
Refractive index detector temperature	40°C
Injection volume	20 *µ*L
Pressure	60 bar

**Table 2:** Physical compositional characteristics of cashew apple juice.

Cashew growth regions	Physical characteristics		References
	Specific gravity	pH	
South Africa, Africa	1.050	4.52	This study
Nigeria, Africa	1.046	4.15	Akinwale [13]
Ghana, Africa	—	4.08–4.59	Lowor and Agyente-Badu [19]
Benin, Africa	—	3.85–4.02	Michodjehoun-Mestres et al. [17]
Bengal, India	1.069	3.90–4.14	Attri [20]
Ariyalur District, India	—	4.86–5.54	Sivagurunathan et al. [21]
Ceara State, Brazil	—	4.00–4.60	Michodjehoun-Mestres et al. [17]
Rio de Janeiro, Brazil	—	3.77–3.91	Campos et al. [22]
Sao Paulo, Brazil	—	3.50–4.50	Assunção and Mercadante [8]

**Table 3:** Chemical compositional characteristics of cashew apple juice.

Cashew growth regions	Characteristics						References
	Fructose g/L	Glucose g/L	Condensed tannins mg/L	Vitamin C mg/100 mL	Total minerals ppm	Total protein g/L	
South Africa, Africa	57.06 ± 0.30	40.56 ± 0.32	55.34 ± 0.003	112 mg/100 mL ± 0.58	15.89 ± 0.12	1.78 ± 0.01	This study
Nigeria, Africa	—	—	—	203.50	—	—	Akinwale [13]
Ghana, Africa	—	—	145.30–306.40	206.20–268.6	0.2–167	—	Lowor and Agyente-Badu [19]
Bengal, India	—	—	—	209.76	—	—	Attri [20]
Fortaleza, Brazil	37.46–56.00	43.28–46.34	—	—	—	2.58	Chagas et al. [23]; Honorato et al. [24] Luz et al. [25]; Rabelo et al. [26]; Honorato and Rodrigues [27];
Sao Paulo, Brazil	—	—	—	104–121	—	—	Assunção and Mercadante [8]
